# FTR83, a Member of the Large Fish-Specific finTRIM Family, Triggers IFN Pathway and Counters Viral Infection

**DOI:** 10.3389/fimmu.2017.00617

**Published:** 2017-05-26

**Authors:** Christelle Langevin, Elina Aleksejeva, Armel Houel, Valérie Briolat, Corinne Torhy, Aurélie Lunazzi, Jean-Pierre Levraud, Pierre Boudinot

**Affiliations:** ^1^INRA, Virologie et Immunologie Moléculaires, Jouy-en-Josas, France; ^2^Institut Pasteur, Unité Macrophages et Développement de l’Immunité, Paris, France; ^3^CNRS, URA 2578, Paris, France

**Keywords:** FTR83, finTRIM, interferon, antiviral immunity, zebrafish

## Abstract

Tripartite motif (TRIM) proteins are involved in various cellular functions and constitute key factors of the antiviral innate immune response. TRIM proteins can bind viral particles directly, sending them to degradation by the proteasome, or ubiquitinate signaling molecules leading to upregulation of innate immunity. TRIM proteins are present in across metazoans but are particularly numerous in vertebrates where genes comprising a B30.2 domain have been often duplicated. In fish, a TRIM subset named finTRIM is highly diversified, with large gene numbers and clear signatures of positive selection in the B30.2 domain suggesting they may be involved in antiviral mechanisms. finTRIM provides a beautiful model to investigate the primordial implication of B30.2 TRIM subsets in the arsenal of vertebrate antiviral defenses. We show here that *ftr83*, a zebrafish *fintrim* gene mainly expressed in the gills, skin and pharynx, encodes a protein affording a potent antiviral activity. *In vitro*, overexpression of FTR83, but not of its close relative FTR82, induced IFN and IFN-stimulated gene expression and afforded protection against different enveloped and non-enveloped RNA viruses. The kinetics of IFN induction paralleled the development of the antiviral activity, which was abolished by a dominant negative IRF3 mutant. In the context of a viral infection, FTR83 potentiated the IFN response. Expression of chimeric proteins in which the B30.2 domain of FTR83 and the non-protective FTR82 had been exchanged, showed that IFN upregulation and antiviral activity requires both the Ring/BBox/Coiled coil domain (supporting E3 ubiquitin ligase) and the B30.2 domain of FTR83. Finally, loss of function experiments in zebrafish embryos confirms that ftr83 mediates antiviral activity *in vivo*. Our results show that a member of the largest TRIM subset observed in fish upregulates type I IFN response and afford protection against viral infections, supporting that TRIMs are key antiviral factors across vertebrates.

## Introduction

Upon pathogen invasion, host immune response starts with detection of microbial products by pattern recognition receptor (PRR), leading to cell activation and synthesis of inflammatory cytokines. The innate immune response to viruses involves several dedicated toll-like receptors (TLR) and RIGI-like receptors, which trigger two main signaling pathways—IKKα/β-NFkB and IKKε/TBK1-IRF3/7—and the synthesis of type I IFN (1). Type I IFN can function in a paracrine or autocrine manner; upon binding to their receptor at the cell surface, signal transduction through the Jak/STAT pathway leads to the formation of the ISGF3 complex [reviewed in Ref. (2)]. ISGF3 then translocates to the nucleus and promotes the expression of many effector interferon induced genes (ISG), some of which have antiviral activity. This virus-induced response is tightly regulated by multiple mechanisms, including positive and negative feedback loops mediated by ISG. In contrast, other antiviral factors can be intrinsically expressed like APOBEC3G and TRIM5α (3), interfering at different steps of the virus cycle.

Many tripartite motif (TRIM) proteins are important antiviral factors involved in antiviral defense *via* multiple mechanisms: as direct effectors often induced by type I IFN, or as modulators/enhancers of the response (4). TRIM proteins are defined by their Ring/BBox/Coiled coil (RBCC) TRIM. The really interesting new gene (RING) domain has an E3 ligase activity promoting conjugation of Ub or Ub-like residues such as SUMO and ISG15 to target proteins (5); BBox is another type of Cys-based motif, and Coil-coiled domains are involved in TRIM proteins homo-dimerization or oligomerization into large complexes (6). The RBCC motif is generally followed by a C-terminal domain, which is highly variable and determines subcellular localization, interactions with other proteins and cellular functions of the protein. For example, the B30.2 domain—also known as “PRY-SPRY” domain (7), is found in many TRIM with antiviral functions including TRIM5, TRIM21, TRIM22, and TRIM25. This domain constitutes a versatile scaffold promoting the assembly of protein complexes (8, 9) and may also have RNA binding properties (10).

Tripartite motif can be involved in direct antiviral mechanisms, in control of viral gene transcription, or in the regulation of the innate immune response. TRIM5α is one of the best-studied members of the family with a direct antiviral activity, triggered by B30.2 binding to HIV1 capsid shortly after virus entry into the cell (11). TRIM5α oligomers thus cover the viral capsid, and the complex is degraded by the proteasome through a process dependent of TRIM5α auto-ubiquitination (12). In addition, TRIM5α is a potent modulator of antiviral innate immunity (13). TRIM22 is another protein exerting its antiviral activity through direct interaction with proteins from different viruses, blocking their trafficking and promoting their degradation *via* ubiquitination (14, 15). B30.2 domains of TRIM5 and TRIM22 evolved under strong positive selection, underlining the importance of this domain as a specific recognition module check (16). TRIM21 also acts through a direct mechanism, targeting non-enveloped antibody-opsonized virus in the cell cytoplasm, and sending them to degradation by the proteasome (17). The recognition of intracytoplasmic antibodies bound to pathogens by TRIM21 B30.2 domain also induces innate immune response and cytokine production (18). Alternatively, TRIM with a bromodomain target histones and mediate chromatin remodeling; TRIM28 thus plays an important role in the silencing of retrovirus genes (19). Finally, TRIM can also play an indirect role in antiviral immunity by modulating immune signaling, mostly *via* ubiquitination of key factors of these pathways. The best characterized of these mechanisms is probably the ubiquitination of the RNA sensor RIG-I by TRIM25, leading to the formation of the MAVS/RIG-I complex and IFN synthesis, and PML/TRIM19 of which one isoform regulates IFN synthesis (20). Importantly, many TRIMs have the capacity of enhancing the innate immune response, through multiple mechanisms: a recent screen showed that about half of all human TRIM proteins affect these signaling pathways (21).

In addition to antiviral immunity, TRIM proteins exert a wide range of cellular functions: as modulators of gene transcription or factors of post-translational modifications *via* their E3 ligase activity, they are implicated in gene expression, apoptosis, cell proliferation and differentiation, cancer, inflammation and auto-immune diseases, etc. (6, 22). TRIMs involved in basic cellular functions often belong to the most ancient subsets, e.g., RBCC-Cos-Fn3-B30.2 TRIM, which were already present in early metazoans (23) and are found both in vertebrates and invertebrates (24). Only a few *trim* genes are generally present in the genome of invertebrates, while the family greatly expanded in vertebrates. *Trim* genes with an RBCC-B30.2 domain organization were particularly prone to amplification (25, 26), and large sets of such genes arose from independent expansions in fish, coelacanth, and tetrapods (25–27). The implication of TRIM in antiviral defense likely was a strong selection pressure toward diversification, but the antiviral functions of these proteins in non-mammalian vertebrates remain poorly characterized.

Fish possess a large repertoire of TRIMs, with several large specific gene expansions including finTRIMs (28), bloodthirsty-related TRIM (TRIM39) and TRIM35 (25, 26). Antiviral functions have been recently reported for several TRIM proteins of the orange spotted grouper in *in vitro* systems: TRIM8 and TRIM32 upregulated genes of the type I IFN system, while viral gene transcription was inhibited in cells overexpressing TRIM39 (29–31). In contrast, TRIM13, TRIM16L, and TRIM62L appear to downregulate the antiviral immune response, promoting nodavirus or iridovirus infections (32–34). FinTRIMs, which constitute the largest TRIM expansion observed in zebrafish, are also likely involved in antiviral defenses. These TRIMs, which have an RBCC-B30.2 domain structure, were initially discovered in salmonids as genes induced by the rhabdovirus VHSV in leukocytes *ex vivo* (35). In the zebrafish, the B30.2 domain of finTRIMs evolved under strong positive selection, at positions remarkably congruent with those identified in the viral recognition motif of TRIM5α in primates (28, 36). Zebrafish functional *ftr* genes are typically expressed at very low basal levels and weakly induced by the viral infection. Most of the 80 *ftr* genes appeared recently, having no “one-to-one” orthologs in other fish families such as salmonids or the pufferfish family (26). Interestingly, two members of the family (*ftr82* and *ftr83*) did not follow this pattern: they had orthologs in the main fish branches (28), indicating that they were likely related to the basal (i.e., ancestral) finTRIM genes. They were expressed at a higher constitutive level in multiple tissues of zebrafish larvae and were not induced by viral infection or IFN treatment. In this work, we investigated the implication of these genes in antiviral immunity. We show that FTR83 significantly increases basal IFN expression and modulates expression of ISGs, mediating a potent antiviral activity against RNA viruses *in vitro* and *in vivo*. In contrast, FTR82—another FTR closely related to FTR83 but with distinct expression pattern, does not possess such properties. Chimeras between FTR82 and FTR83 showed that both FTR83 RBCC and B30.2 are required for antiviral functions. Our data show that positive regulation of the IFN pathway and antiviral functions are a fundamental property of finTRIMs, and more generally of TRIM proteins across vertebrate immune systems.

## Materials and Methods

### Ethics Statement

All animals were handled in strict accordance with good animal practice as defined by the European Union guidelines for the handling of laboratory animals (http://ec.europa.eu/environment/chemicals/lab_animals/home_en.htm) and by the Regional Paris South Ethics committee. All animal work was approved by the Direction of the Veterinary Services of Versailles (authorization number 78-28) as well as fish facilities (authorization number B78-720). Experimental protocols involving zebrafish were approved by the INRA institutional ethical committee “Comethea” (#12/114).

### Primary Antibodies

Anti-HA 3F10 monoclonal antibody was purchased from Roche and anti-V5 monoclonal antibody from Molecular Probes.

### Cloning of *ftr82* and *ftr83*

Ftr82 (ENSDARG00000055647, transcript ENSDART00000016758.7), and ftr83 (ENSDARG00000025403, transcript ENSDART00000098239.4) were cloned in fusion with an HA tag, respectively, in pcDNA3.3 and pcDNA3.1 (Table S1 in Supplementary Material). FTR chimeras were obtained by recombinant PCR using V5-ftr82 and ftr83-HA as templates. RBCC domains of ftr82 and ftr83 were, respectively, amplified with fwFTR82-Attb1/revFTR82-_B30.2ftr83_ and fwFTR83-HA/revFTR83-_B30.2ftr82_. B30.2 domains of ftr82 and ftr83 were, respectively, amplified with fwFTR83-_B30.2ftr82_/revHA-Ftr82, and fwFTR82-_B30.2ftr83_/revFTR83B30.2-Attb2-nostop. These PCR products were then annealed, and the full constructs were amplified with fwFTR82-Attb1 and revFTR83B30.2-Attb2-nostop for 82-83, and with fwFTR83-HA and revHA-Ftr82 for 83-82. 82-83 was cloned with the Gateway cloning system (Invitrogen) in pDSET 6.2V5 to be expressed with V5 tag fused to the C terminus. 83-82 was cloned with the TOPO TA cloning system (Invitrogen) in pcDNA3.3 to be expressed with HA tag fused to the N terminus. *Ftr82* and *ftr83* coding regions were also amplified using primers HA-ftr83 and ftr82-HAftr82-Attb1, ftr83-Attb1, and ftr82Attb2nostop, ftr83Attb2nostop (Table S1 in Supplementary Material), cloned into the entry vector of the Gateway cloning system (Invitrogen) then transferred to the different destination vectors. Ftr82 was transferred in pDEST6.2-V5 or pDSET 47 to be expressed with V5 or GFP tag fused to the C terminus. Ftr83 was transferred to pDEST53 to be expressed with GFP tag fused to the N terminus. *Ftr83* deletion mutants were obtained using specific primers on ftr83-HA template. Ftr83B30.2 was constructed with Ftr83B30.2-Attb1 and Attb-2-nostop primers while FTR83ΔB30.2 was constructed with FTR83ΔB30.2-Attb1 and Attb2 primers (Table S1 in Supplementary Material). PCR products were then cloned using the Gateway cloning system in pDSET 47 to be expressed with GFP tag fused to the C terminus.

### Whole Mount *In Situ* Hybridization

Whole mount *in situ* hybridization was performed as described in Ref. (37), using NBT/BCIP revelation (Sigma). Antisense probes for ftr82 (product size 856 bp) and ftr83 (product size 865 bp) were generated with T3 polymerase (Promega). Templates for *in vitro* transcription were amplified using primers shown in Table S1 in Supplementary Material, and PCR products were purified using Microspin™ S-400 HR columns (GE Healthcare).

### Fish, Cells, and Viruses

Zebrafish were raised in the fish facilities of Institut National de la Recherche Agronomique (Jouy-en-Josas, France). Epithelioma papulosum cyprini (EPC) cell line (ATCC^®^ CRL-2872™) was maintained in Glasgow’s modified Eagle’s medium-HEPES 25 mM medium (Eurobio) supplemented with 10% fetal bovine serum (FBS, Eurobio, produced and distributed in France under the veterinary authorization FR 91 692 200), 1% tryptose phosphate broth (Eurobio), 2mM l-glutamine (PAA) and antibiotics 100 µg/mL penicillin (Biovalley), 100 µg/mL streptomycin (Biovalley). Transfection experiments, viral production, and titration were performed in EPC cells. The novirhabdoviruses infectious hematopoietic necrosis virus 32-87 (IHNV) and viral hemorrhagic septicemia virus 07-71 (VHSV) and the vesiculovirus spring viremia of carp virus (SVCV) were produced at 14°C on EPC in GMEM media supplemented with 2% FBS, 5% tryptose, and 2mM l-glutamine. Cytopathic effect was evaluated 72 h postinfection after cell fixation with 10% formol prior to coloration in 2% crystal violet.

### Transfection

Epithelioma papulosum cyprini cells were nucleotransfected with the nucleofector kit T (Lonza) following the manufacturer’s recommendations. Briefly, 4 × 10^6^ EPC cells were plated in P6 wells. The day after, cells were trypsinized, resuspended in 100 µL of nucleofector solution with 3–5 µg of DNA. After nucleotransfection, cells were resuspended in a P6 well plate for RTQPCR analyses or immunocytochemistry on PDL (10 µg/mL) coated glass coverslips. Viral challenge was performed on P24 wells seeded with one million of transfected cells 24 h before viral infections.

### Morpholino Knockdown Experiments

Experiments were performed as described in Ref. (38). Eggs of wild-type AB zebrafish (one-cell stage) were microinjected with 4 ng of morpholinos (MO) (Gene Tools): Ftr83-specific MO (MOftr83: TTACAACTAGACTACATACCTGTCT); Ftr82-specific MO [MOftr82: GCGCTATGTTTTCCTTACCTGTTTT from Ref. (39)]; or control morpholino with no known target (MOctl: GAAAGCATGGCATCTGGATCATCGA). Ftr82- and ftr83-specific MO target splice donor sites, and efficiency of knockdown was assessed by qRT-PCR with splice-sensitive primers (Table S1 in Supplementary Material). Embryos then developed with no obvious morphological defects at 28°C until 72 hpf. Anesthetized 72 hpf larvae were challenged by intravenous inoculation of 100 pfu of SVCV (38) or left untreated, 6 h postinfection, embryos were collected for gene expression analysis by RT-QPCR.

### *In Vitro* Infections

Ninety-six hours post transfection (hpt), EPC cell monolayers were infected with rhabdoviruses MOI1 by a 1 h absorption step at 14°C in GMEM 2% FBS. After removal of the inoculum, cells were incubated in GMEM 2% FBS at 14°C for the rest of the experiment. Cell supernatant was taken postabsorption and after 8, 24, 48, and 72 h of infection for virus titration experiments. Infected cells were fixed at 72 hpi to evaluate cytopathic effect by crystal violet coloration. Short SVCV infections were performed at 72 hpt. Cells were exposed to virus inoculum (MOI1) 6 h at 14°C before analysis of gene expression. EPC cells were also infected with the birnaviruses IPNV strains VR299 or 31-75, in GMEM media supplemented with 2% FBS, 5% tryptose, and 2 mM l-glutamine. Cytopathic effect was evaluated 72 h postinfection after cell fixation with 10% formol prior to coloration in 2% crystal violet. The birnaviruses were propagated on BF cells at 14°C. Viruses were titrated on EPC by plaque assay as previously described in Ref. (40).

### RNA Isolation, cDNA Synthesis

Total RNA extraction was performed by TRIZOL (Invitrogen) from four million EPC cells at 72 hpt or from zebrafish tissues sampled from 2- to 3-month-old zebrafish (strain AB). RNA was purified using the RNeasy mini kit (QIAGEN) according to the manufacturer’s instructions and treated with DNAse. Reverse transcription experiment was performed on 1 µg of total RNA using 125 ng of random hexamer primers (Roche) in a Superscript II Reverse transcriptase kit (Invitrogen) according to the manufacturer’s instructions.

### Real-time Q-PCR

Gene expression was measured by real time PCR with a Realplex^2^ Mastercycler Instrument (Eppendorf) using Power SYBR^®^ Green PCR Mastermix (Applied Biosystems). Each sample is composed by 5 µL of primers (300 nM each), 5 µL of cDNA (diluted 1/10 for cell samples and 1/5 for zebrafish samples), and 10 µL of PCR Mastermix. Samples were first incubated for 2 min at 50°C and for 10 min at 95°C, then subjected to 40 amplification cycles (95°C for 15 and 60°C for 1 min), followed by 15 s at 95°C, 15 s at 60°C, 20 min from 60 to 95°C and finally 15 s at 95°C, to establish the melting curve of PCR products. Gene expressions were computed according to the ABI Prism 7700 user bulletin (Applied Biosystems) and normalized to the beta-actin expression level. All QPCR primers used in this study are shown in Table S1 in Supplementary Material.

### Immunocytochemistry

Seventy-two hours post transfection, cells were fixed in PBS pH7.4 PFA 4% for 20 min at 4°C. After fixation, cells were permeabilized in 0.2% Triton X100 solution for 5 min at RT before saturation in PBS 2% BSA solution at RT for 1 h. Cells were then incubated with anti-HA (Roche) or anti-V5 (Invitrogen) monoclonal antibodies in PBS, 2% BSA, 0.1% Triton X100 for 1 h. Primary antibody binding sites were then revealed by incubation with Alexa coupled secondary antibodies (Molecular Probes) in PBS, 2% BSA, 0.1% Triton X100, and DAPI for 1 h at RT before mounting in Immuno Mount solution (Molecular Probes). Images were acquired on AxioObserver Z1 microscope (Zeiss) with a 63× Plan Neofluar objective using Photometrics CoolSNAP HQ2 Camera.

### Homology Modeling

Structural models of FTR82 and FTR83 B30.2 domains were built with Swiss-model program (41) using the high-resolution structure of mammalian TRIM25 as a template (PDB 4B8E). Chimera program was used for structure viewing and ftr B30.2 superposition (42).

## Results

### *ftr82* and *ftr83* Are Archetypal Members of the Large Fish Multigenic *ftr* Family

**Figure 1:**
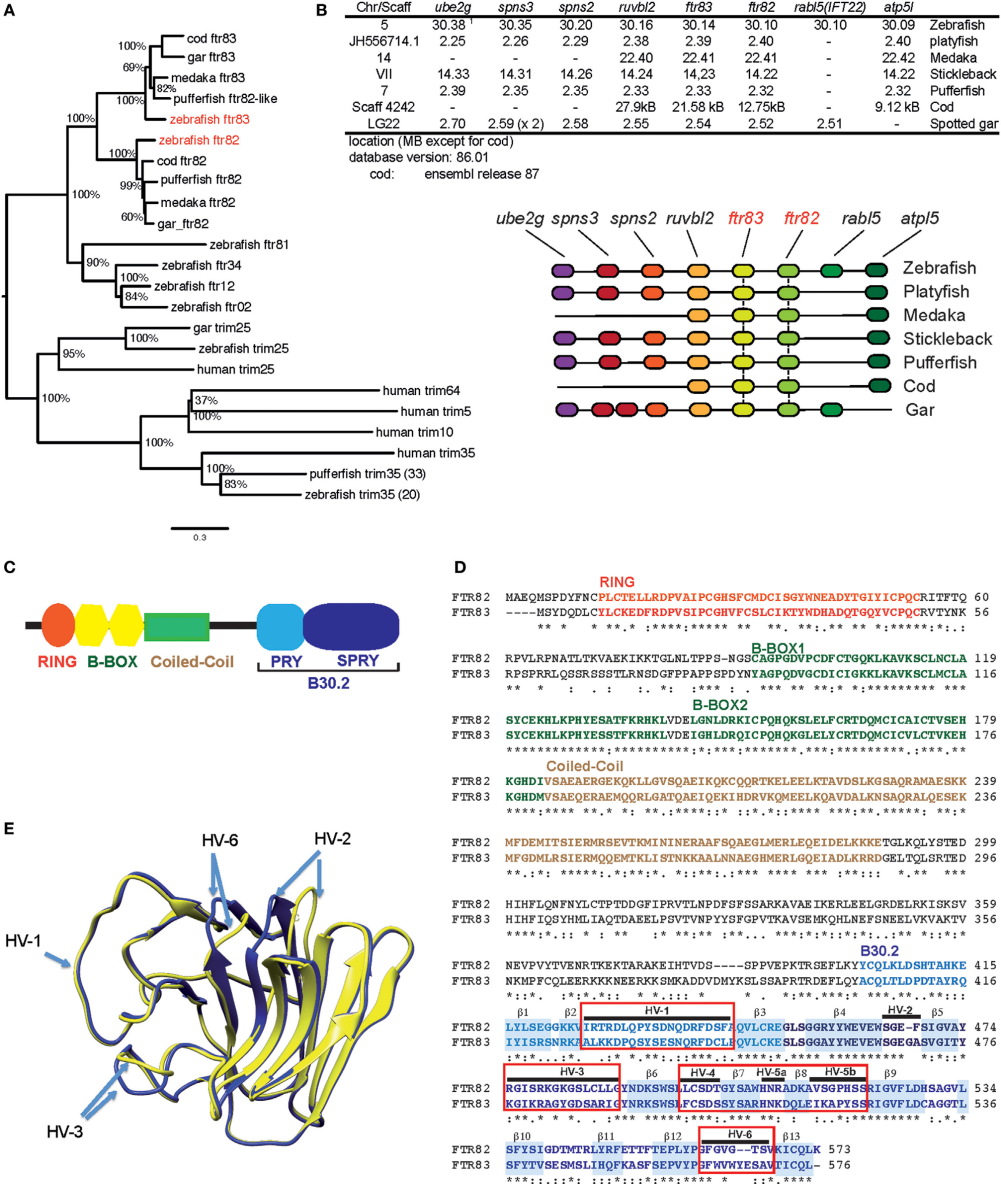
***ftr82* and *ftr83*, two members of the fintrim family conserved across teleost fish**. **(A)** Neighbor joining tree including zebrafish *ftr82* and *ftr83*, and their orthologs in several fish species, other fintrims, and the most similar homologs of finTRIM in fish and in human. **(B)** Comparison of the conserved genomic context of *ftr82* and *ftr83* genes in different fish species, as shown using the Genomicus software, with their genomic location. **(C)** Typical domain structure of finTRIMs. **(D)** Alignment of FTR82 and FTR83 sequences with hyper variable loops of the B30.2 domain highlighted. **(E)** Molecular modeling of B30.2 domains from FTR82 (yellow) and FTR83 (blue) derived from homology modeling based on crystal structure of huTRIM25 B30.2 domain. Visualization of models superposition was performed with the program Chimera.

The *fintrim (ftr)* family extensively diversified in parallel in each fish lineage (26). Among the 80 genes found in the zebrafish, transcriptome studies (43) showed that, unlike most other *ftr, ftr82*, and *ftr83* were well expressed constitutively in the larva, but not induced by IFN or viral infection (Figure S1 in Supplementary Material). Another feature that sets these genes apart from the other *ftr* is that they possess true orthologs in other fish species. Phylogenetic analysis of FTR sequences from different fish shows that both FTR82 and FTR83 sequences cluster in a well-supported group, as illustrated in Figure 1A. In contrast, other FTR clusters comprise sequences correspond to more recent, lineage-specific diversification (26). Within the conserved FTR82/83 group, FTR82 and FTR83 each define a specific set of orthologs; both ftr82 and ftr83 have an ortholog in a holostean fish, the spotted gar, indicating that *ftr82* and *ftr83* ancestors were already present before the divergence of modern groups of fishes. *ftr82* and *ftr83* are part of a synteny group conserved between cypriniformes (zebrafish), percomorphs (medaka, stickleback, platyfish, and pufferfish), gadiforms (cod), and holosteans (spotted gar), supporting the idea that they resulted from a local duplication of a common ancestor that occurred prior to teleost radiation (Figure 1B).

FTR82 and FTR83 have the typical domain structure of finTRIMs, comprising a RING/B-Box/Coiled coil TRIM and a typical B30.2 domain (Figure 1C). Zebrafish FTR82 and FTR83 protein sequences are 55% similar to each other (Figure 1D), but only 35–45% similar to other zebrafish FTRs (not shown). Hypervariable loops of the B30.2 domains are not highly divergent between FTR82 and FTR83 (Figures 1D,E), in contrast to what is observed for the whole FTR group (28).

These observations indicate that *ftr82* and *ftr83* are “ancient” *ftrs* with structure and genomic context conserved across fishes and that they may have a generic function different from the main set of *ftr*, which repeatedly diversified during fish evolution.

### *ftr83* Is Mainly Expressed in Tissues Exposed to the Environment and in Hematopoietic Tissues, while *ftr82* Has a Wider Expression Range

**Figure 2:**
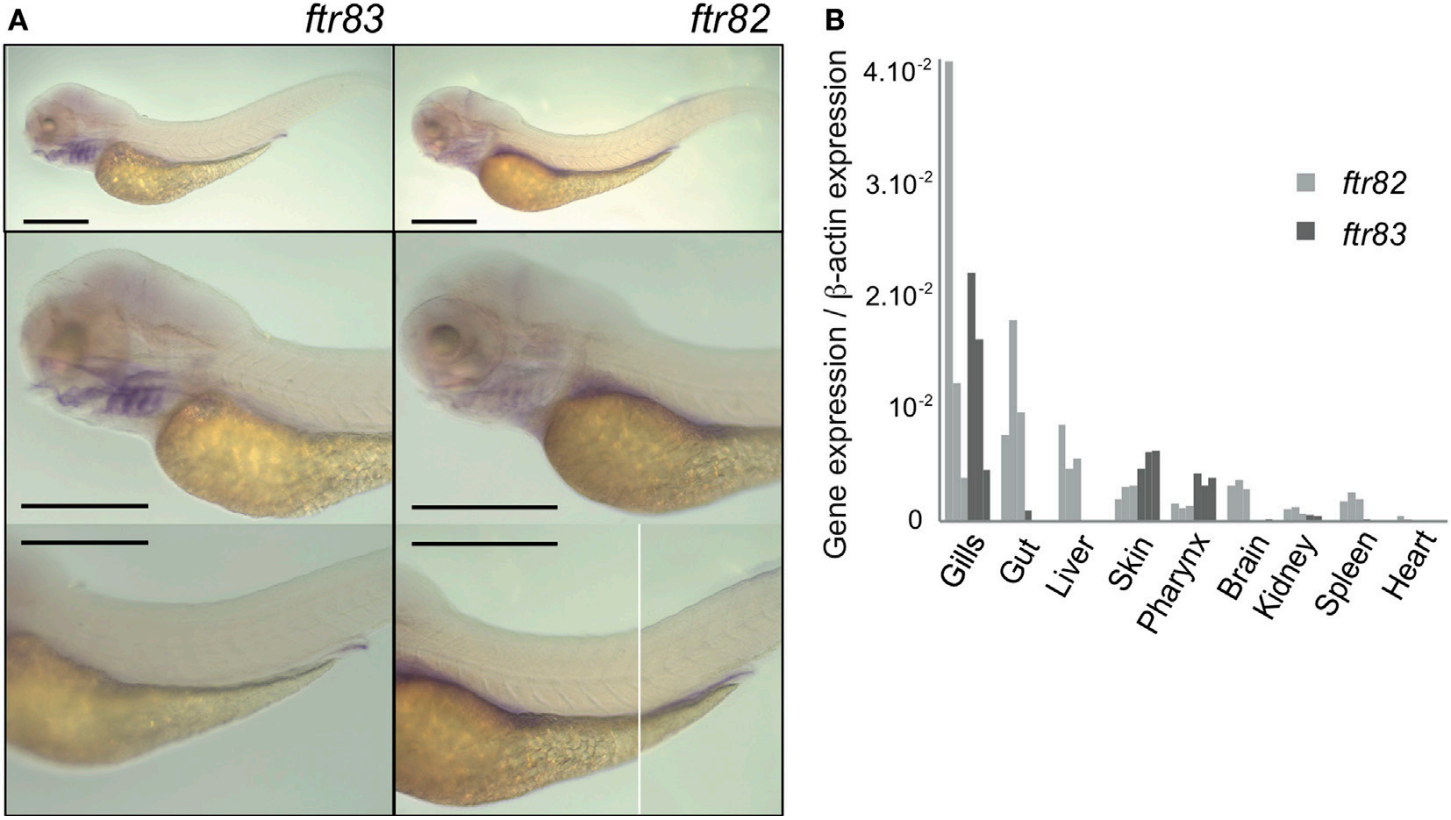
***ftr82 and ftr83* expression pattern are distinct**. **(A)** Spatial expression of *ftr83* and *ftr82* in 3.5 dpf zebrafish larvae. WISH using antisense probes indicated on each panel. Scale bars: 0.5 mm. **(B)** Genes expression in 2–3 months old juvenile zebrafish, measured by RTQPCR in various dissected tissues. Transcript copy numbers were normalized to β-actin expression: measured ratio of cDNA of interest/β-actin cDNA is shown. Results of three biological replicates, each being a pool of organs from 15 fish.

To determine the spatial pattern of expression of *ftr82* and *ftr83*, we first used whole mount *in situ* hybridization in zebrafish larvae. Figure 2A shows that *ftr82* and *ftr83* are both well detectable but have distinct tissue distributions at 3.5 days postfertilization (dpf): while *ftr82* has a relatively wide range of expression with high levels in the gut, *ftr83* is more restricted to the pronephric duct and pharyngeal area, notably gill arches. These patterns are fully consistent with those reported in the public ZFIN database at the high/long-pec stage (i.e., ~2 dpf) [see, for *ftr82*, http://zfin.org/ZDB-IMAGE-021210-600 (44, 45); and for *ftr83*, http://zfin.org/ZDB-FIG-050630-7888 (45), and do not contradict the *ftr82* pattern reported in younger embryos (30 hpf) by Chang et al. (39)]. In addition, they persist in the young adult, as shown by real time QPCR data from isolated organs of 3-month zebrafish (Figure 2B). At this stage, *ftr83* expression is mainly observed in gills, skin, and pharynx; it can also be detected in hematopoietic tissues (spleen and kidney) although at a much lower level. In contrast, *ftr82* was well expressed in many tissues without clear tropism*. In situ* hybridization profiles also clearly indicate that in larvae, *ftr82* and *ftr83* are not primarily expressed in leukocytes, which would result in a punctate pattern.

Strikingly, while *ftr82* and *ftr83* were expressed in multiple tissues at a fair level, and more than other *ftrs*, they were not induced by IFN (Figure S1 in Supplementary Material).

Altogether, these data confirm that *ftr82* and *ftr83* do not constitute typical ISG, in contrast to other finTRIMs that are generally expressed at very low level in tissues of healthy fish and can be induced by viral infection and IFN (26, 35). We therefore hypothesized that these two conserved TRIMs expressed at steady-state might be involved in the natural antiviral immunity.

### FTR83 Affords Protection against RNA Viruses *In Vitro*

**Figure 3:**
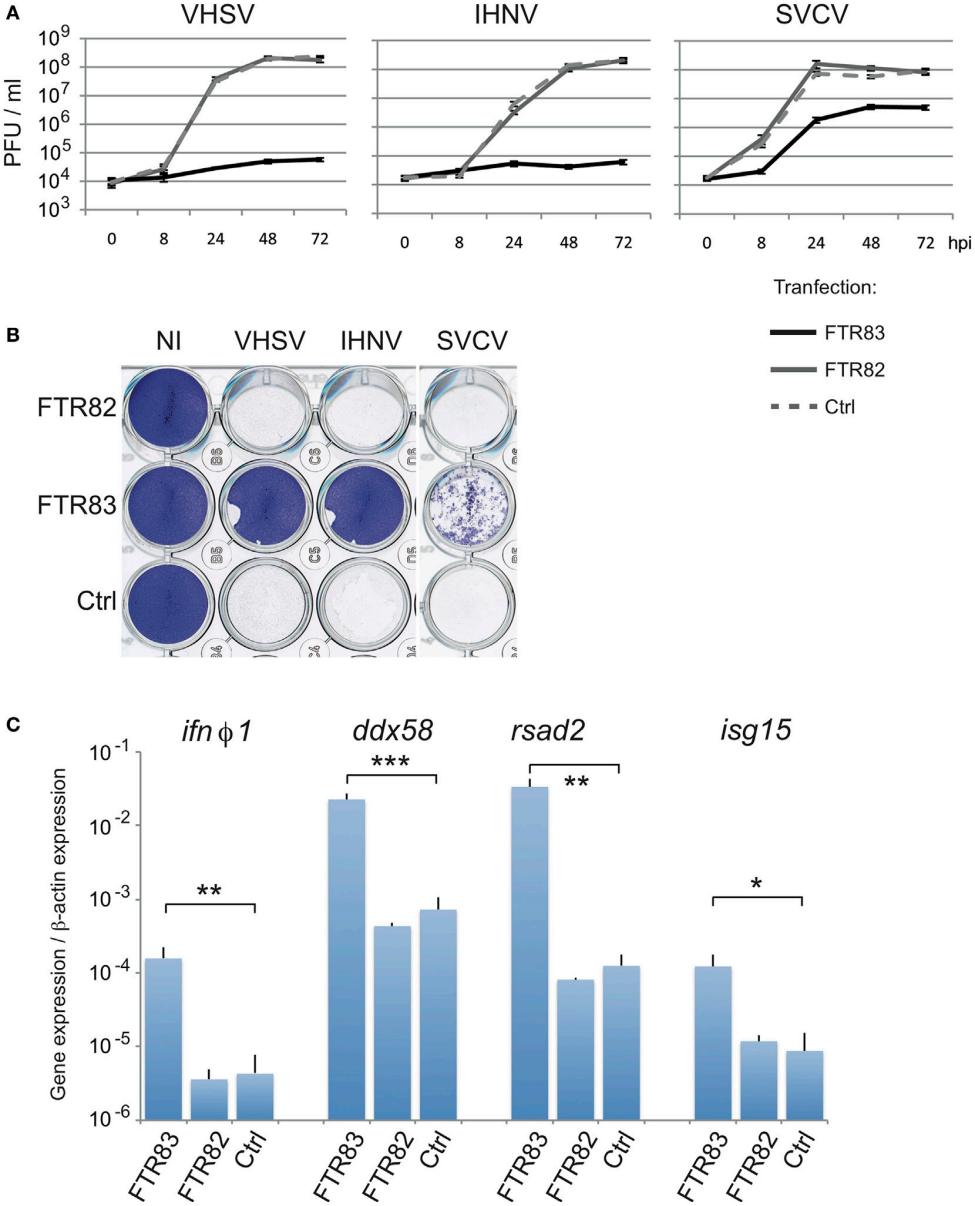
**FTR83 overexpression inhibits viral infection**. **(A)** Kinetic of viral growth measured by viral titration from 0 to 72 hpi in the supernatants of cells transfected with expression plasmids for *ftr83* (black line) or *ftr82* (gray line) or with empty vector as control (dotted line). Cells were infected at 72 hpt with IHNV, VSHV, or spring viremia of carp virus (SVCV) (MOI 1). The mean and SD of three independent experiments are presented. **(B)** Cytopathic effect of viral infections. Cells were infected 72 h post transfection at MOI1 and viral-induced cytopathic effect was assessed by crystal violet staining at 72 hpi. Non-infected cells are presented as a control (NI). **(C)** FTR83 triggers the IFN signaling pathway. Epithelioma papulosum cyprini cells were transfected with expression vectors for *ftr82, ftr83* or with empty plasmid (Ctrl) and analyzed 72 h post transfection for modulation of genes of the IFN pathway: *ifn*ϕ*1, ddx58, rsad2, isg15*. RTQPCR results were normalized on the β-actin expression: measured ratio of cDNA of interest/β-actin cDNA is shown. Mean and SD are shown, for three independent experiments; stars indicate significant differences using Student’s *t*-test (***p < 0.001, **p < 0.01, *: *p* < 0.05).

To investigate whether *ftr82* and *ftr83* might contribute to antiviral defense, we assessed the impact of FTR83 (and FTR82) overexpression on the susceptibility to several viral infections. To this purpose, we first used a well-established fish cellular model (40). Seventy-two hours post transfection, EPC cells were exposed to distinct viruses (MOI1), and inhibition of viral growth was evaluated in titration experiments from cell supernatant up to three days postinfection. Figure 3A shows the effect of expression of the two HA-tagged FTRs on growth kinetics of SVCV, IHNV, and VSHV. FTR83 overexpression drastically inhibited viral growth for both IHNV and VHSV, reducing viral titers about 3,000-fold compared to FTR82 or to mock-transfected cells, at 72 hpi. The inhibition of SVCV was less efficient, but still highly significant with a 15 fold difference of virus titers between FTR83 and other conditions over the same period. The protection afforded by FTR83 overexpression against VHSV and IHNV was also observed at MOI3 (Figure S2A in Supplementary Material).

Accordingly, overexpression of FTR83 prevented viral-induced cytotoxicity and efficiently preserved the integrity of the cell monolayer after infection with IHNV, VHSV, or SVCV, as demonstrated by crystal violet colorations (Figure 3B). In contrast, cytopathic effect of viral infections led to the complete destruction of the cell monolayer at 72 hpi upon FTR82 expression or in mock-transfected conditions (Figure 3B). These observations were extended to non-enveloped viruses, as FTR83 overexpression fully protected the cell monolayer against two strains of the birnavirus IPNV (Figure S2B in Supplementary Material).

We extended our observations to other *ftr* fusion proteins, replacing HA tag by V5 or GFP for FTR82, and by GFP for FTR83, in N-ter or C-ter position. While all versions of FTR83 afford robust protection from IHNV and VHSV, none of the FTR82 constructs showed a significant antiviral effect (Figures S2C,D in Supplementary Material: compare cells transfected with FT82 plasmids with cells transfected with a control plasmid or with non-transfected cells), indicating that the effect did not depend on the tag or its position.

Altogether, these results show that FTR83—but not FTR82—confers a potent resistance against several enveloped or non-enveloped RNA viruses, suggesting that this TRIM triggers a generic antiviral mechanism.

### FTR83 Promotes the Expression of Key Components of the IFN Pathway

The range of virus efficiently contained by FTR83 pointed to a general mechanism, possibly linked to the type I IFN response. We therefore studied the impact of FTR83 overexpression on genes involved at different levels of the IFN/PRR signaling pathway in fish and mammals (Figure 3C), including the molecular sensor *ddx58* (also known as *rig-I*); several kinases (*tbk1, ralbp1* also known as *rip1*, and *jak1*); key transcription factors as interferon regulatory factors [*irf3, 7*, and *9*; Signal Transducers and Activators of Transcription (*stat*) *1a, 1b*, and *2*]; type I interferon itself (*ifn*ϕ*1*) and its receptor *crfb5*, as well as two ISGs: *isg15* and *rsad2*. TLR signaling was also investigated through adaptor molecules (*Myd88* and *ticam1*).

The expression of these genes was measured by real time QPCR from EPC fish cells overexpressing HA-tagged FTR82 or FTR83 proteins 72 h post transfection. Strikingly, *ifn*ϕ*1* and the known ISGs *rsad2, isg15, ddx58/rigI, irf7*, and *stat1b* were significantly upregulated upon expression of FTR83, but not FTR82, in absence of additional stimulation (Figure 3C; Figures S3A,B in Supplementary Material). Overall, these observations indicate that FTR83 overexpression can trigger IFNϕ1 production and the induction of ISGs, providing a convincing explanation for its antiviral activity.

### A Robust FTR83-Mediated Type I IFN Induction Parallels Cell Protection against RNA Viruses, Requires a Functional IRF3, and Is Observed in the Context of Infection in spite of Reduced Virus Load

To further connect FTR83 antiviral effect to its impact on the IFN pathway, we monitored the kinetics of establishment of the antiviral state after *ftr83* transfection.

**Figure 4:**
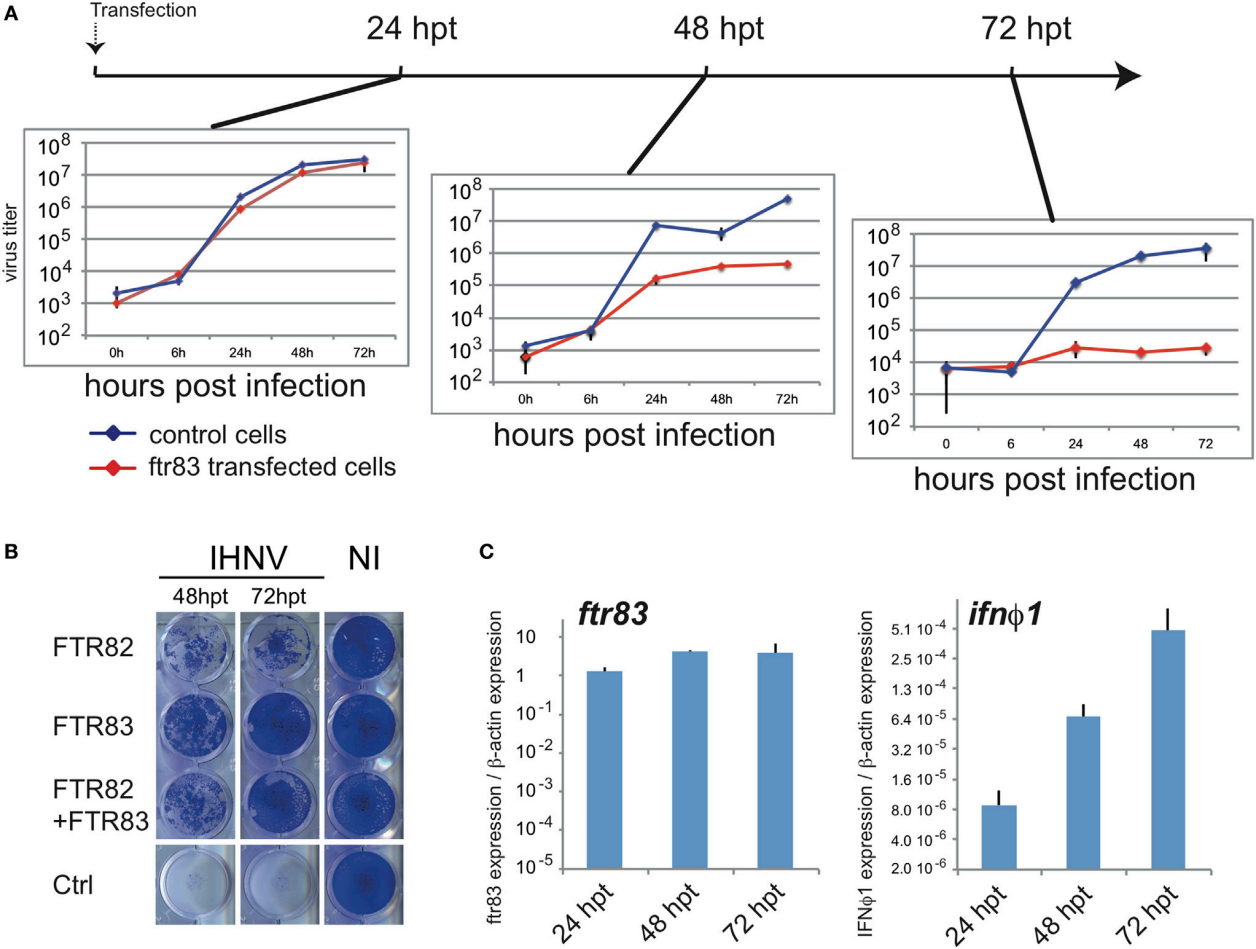
**Antiviral effect of FTR83 is paralleled by stimulation of the type I IFN pathway**. **(A)** Epithelioma papulosum cyprini cells were transfected with *ftr83*-encoding or empty plasmid and were infected with IHNV (MOI1) 24, 48, or 72 h post transfection. Kinetic of IHNV growth was measured by viral titration from 0 to 72 hpi. The mean and SD of three replicates are presented. **(B)** Cytopathic effect of IHNV infections when cells were infected 48 and 72 h post transfection at MOI1. Viral-induced cytopathic effect was assessed by crystal violet staining at 72 hpi. Non-infected cells are presented as a control (NI). **(C)** *ftr83* and *ifn*ϕ*1* transcripts quantified by RTQPCR at the onset of infection, i.e., 24, 48, or 72 h post transfection. Results were normalized on the β-actin expression: measured ratio of cDNA of interest/β-actin cDNA is shown. Mean and SD of three independent experiments.

*Ftr83*-transfected cells were exposed to IHNV (MOI1) at 24, 48, and 72 hpt (hours post transfection) (Figure 4A). Viral inhibition was then monitored by titration experiments from cell supernatant up to 3 days postinfection. Titration curves established from cells exposed to virus inoculum at different times post transfection revealed that IHNV restriction was gradually established from 24 to 72 h post transfection (Figure 4A). While *ftr83* overexpression had no significant effect on IHNV growth at 24 h post transfection, a 2-log decrease in virus titer was observed 48 h post transfection in *ftr83* overexpressing cells, compared to control. This effect was even stronger 72 h post-transfection, *ftr83* triggering a 3-log reduction of virus titer. Accordingly, evaluation of viral-induced cytopathic effect over the same period showed a mild protection 48 hpt compared to control, while a full protection was observed 72 hpt (Figure 4B). In parallel, RTQPCR analyses showed increasing levels of *ifn*φ*1* gene expression from 24 to 72 hpt, while *ftr83* level remained stable over this period (Figure 4C). Altogether, these results show a good correlation between type I IFN expression and protection against IHNV infection.

**Figure 5:**
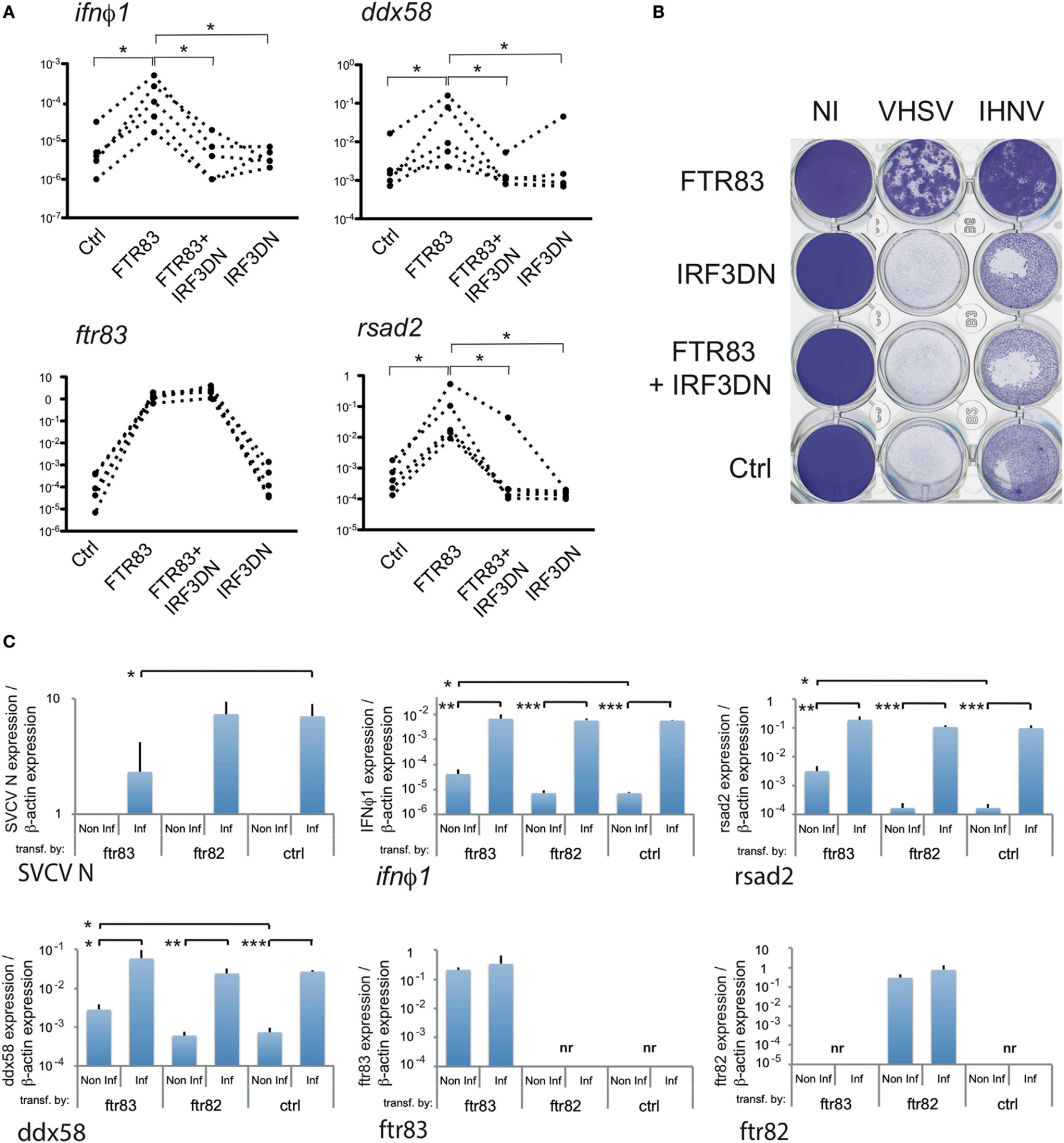
**FTR83-mediated protection against RNA viruses relies on induction of type I interferon**. **(A)** Epithelioma papulosum cyprini cells were transfected with expression plasmid for *ftr83, irf3DN* or co-transfected (*ftr83* + *irf3DN*). Cells transfected with empty plasmid were used as control (Ctrl). Transcripts of interest were quantified by RTQPCR. Five representative experiments are represented, and correspond to dotted lines (*p < 5%, Wilcoxon signed-rank test). **(B)** Transfected cells were infected by IHNV or VHSV (MOI 1) at 72 hpt, and viral-induced cytopathic effect was assessed by crystal violet staining at 72 hpi. Mock-transfected cells (Ctrl) and non-infected cells (NI) were used as controls. **(C)** Transfected cells were infected at 72 h post transfection with spring viremia of carp virus (SVCV) (MOI 1), and transcripts of interest were quantified by RTQPCR 6 h postinfection. Results of RTQPCR were normalized on the β-actin expression: measured ratio of cDNA of interest/β-actin cDNA is shown. Mean and SD are shown, for three independent experiments, and the average of induction or repression fold between infected and non-infected cells is shown when relevant. Stars indicate significant differences using Student’s *t*-test (***p < 0.001, **p < 0.01, *p < 0.05).

To further investigate at what level of the IFN pathway FTR83 was implicated, we analyzed the effect, of a dominant negative mutant of IRF3 (IRF3DN). This mutant consisting of the C-terminal domain of the protein (46) was co-expressed with FTR83 in EPC cells. As IRF3 is a central mediator of type I IFN induction, it was a good candidate to test at which level FTR83 activated the pathway. Of note, *irf3* itself is modestly but significantly upregulated in cells overexpressing FTR83 (Figure S3B in Supplementary Material), thus enhancing its effects on *ifn* induction. The induction of *ifn*φ*1, ddx58*, and *rsad2* previously observed upon FTR83 expression were abolished in cells overexpressing both FTR83 and IRF3DN (Figure 5A), indicating that FTR83 triggering of the IFN pathway occurs upstream of IRF3. Strikingly, the protection of the cell monolayer (Figure 5B: compare cells transfected with the control plasmid, with the plasmid encoding FTR83, and with plasmids encoding IRF3DN and FTR83) was abolished by the expression of IRF3DN, supporting the conclusion that FTR83 antiviral mechanisms depend on IRF3 signaling and IFN induction.

Finally, our data indicate that cells overexpressing FTR83 strongly upregulate key factors of the IFN pathway in the context of a viral infection. Six hours postinfection by SVCV, mRNA encoding the N protein of the virus was much less expressed in cells overexpressing FTR83, compared to the controls or to cells expressing FTR82 (Figure 5C, see also Figure 3A). In these conditions, *ifn*φ*1, rsad2*, and *ddx58* mRNAs reach about the same level of expression in cells expressing FTR83, as in the others (Figure 5C), indicating that FTR83 actually yields a robust virus-induced type I IFN response, in spite of a reduced virus load.

### RING and B30.2 Domains Are Required for the FTR83 Antiviral Effect

**Figure 6:**
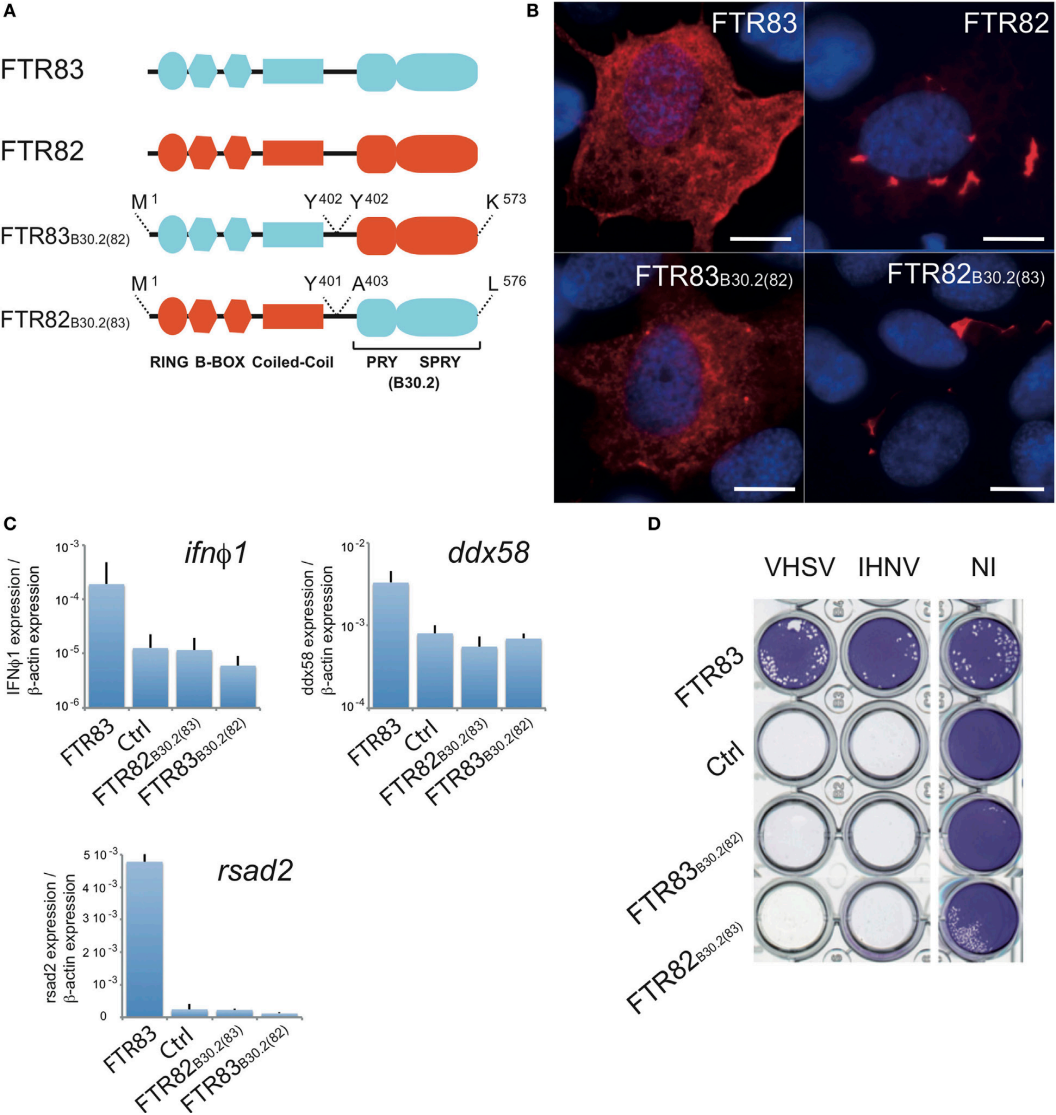
**Ftr83 domains involved in viral restriction**. **(A)** Schematic representations of finTRIM chimeras produced by combination of FTR82 and FTR83 domains. **(B)** Subcellular expression pattern of FTR83, FTR82, and chimeric proteins. HA-tagged FTR83, FTR82, and Ftr83_B30.2(82)_ and V5-tagged Ftr82_B30.2(83)_ were immunostained with relevant antibodies on transiently transfected epithelioma papulosum cyprinid (EPC) cells at 72 hpt. FTR proteins appear in red and nuclei in blue after DAPI staining. Scale bars: 10 µm. **(C)** *Ifn*φ*1, ddx58*, and *rsad2* transcripts are not induced by FTR82_B30.2(83)_ and FTR83_B30.2(82)_ chimeras. Results of RTQPCR were normalized on the β-actin expression: measured ratio of cDNA of interest/β-actin cDNA is shown. Mean and SD are shown, for three independent experiments. **(D)** Cytopathic effect of novirhabdoviruses (IHNV and VHSV) on transiently transfected EPC expressing full length FTR83 (FTR83), FTR chimeras FTR83_B30.2(82)_ or FTR82_B30.2(83)_, or mock-transfected cells (Ctrl). Transfected cells were infected at MOI1 72 hpt, and viral-induced cytopathic effect was assessed by crystal violet staining at 72 hpi. Mock-transfected cells (Ctrl) and non-infected cells (NI) were used as controls.

To identify the domains required for the antiviral effect, we constructed chimeric proteins in which the B30.2 domain had been exchanged between FTR83 and FTR82 as represented in Figure 6A. Expression of both chimeras was investigated by RTQPCR (Figure S4A in Supplementary Material) and immunocytochemistry (Figure 6B). Similar expression was measured at the mRNA level for both chimeras, but distinct intracellular expression patterns were observed, as for wild-type FTR82 and FTR83: FTR83_B30.2(82)_ shows a diffuse pattern with discrete cytoplasmic inclusions as for FTR83, while FTR82_B30.2(83)_ forms large cytoplasmic aggregates as previously observed upon FTR82 overexpression (Figure 6B). The subcellular distribution of FTR82 and 83 thus seems to be mainly determined by their N-terminal (RBCC) domain.

Epithelioma papulosum cyprini cells overexpressing chimeras did not show upregulation of *ifn*ϕ*1, rsad2*, or *ddx58* genes in comparison with mock-transfected cells, indicating that both RBCC and B30.2 domains from FTR83 are required for the induction of the IFN pathway (Figure 6C). Accordingly, no decrease of the IHNV and VHSV cytopathic effect could be observed in cells expressing these chimeras, in contrast to FTR83 (Figure 6D), indicating that both RBCC and B30.2 domains of FTR83 are required for the antiviral function as well. This observation was extended to SVCV infected cells (Figure S4A in Supplementary Material). No impact of chimeras was detected on the expression of the viral *N* transcript, which was consistent with a lack of antiviral activity.

We further designed FTR83 deletion mutants restricted to the B30.2 domain or lacking this domain. Overexpression of these constructs in EPC cells was not sufficient to reduce significantly IHNV or VHSV cytopathic effect (Figures S4C,D in Supplementary Material), supporting the synergistic role of N- and C-term part of the FTR83 protein in the antiviral phenotype.

Altogether, these data indicated that both RBCC and B30.2 of FTR83 are required for innate immunity modulatory effects and antiviral activity, and that neither RBCC nor B30.2 can be substituted by corresponding domains of FTR82. In addition, we did not observe that FTR82 had a dominant negative effect on FTR83 (Figure 4B).

### FTR83 Knockdown Demonstrates FTR83 Antiviral Function *In Vivo*

**Figure 7:**
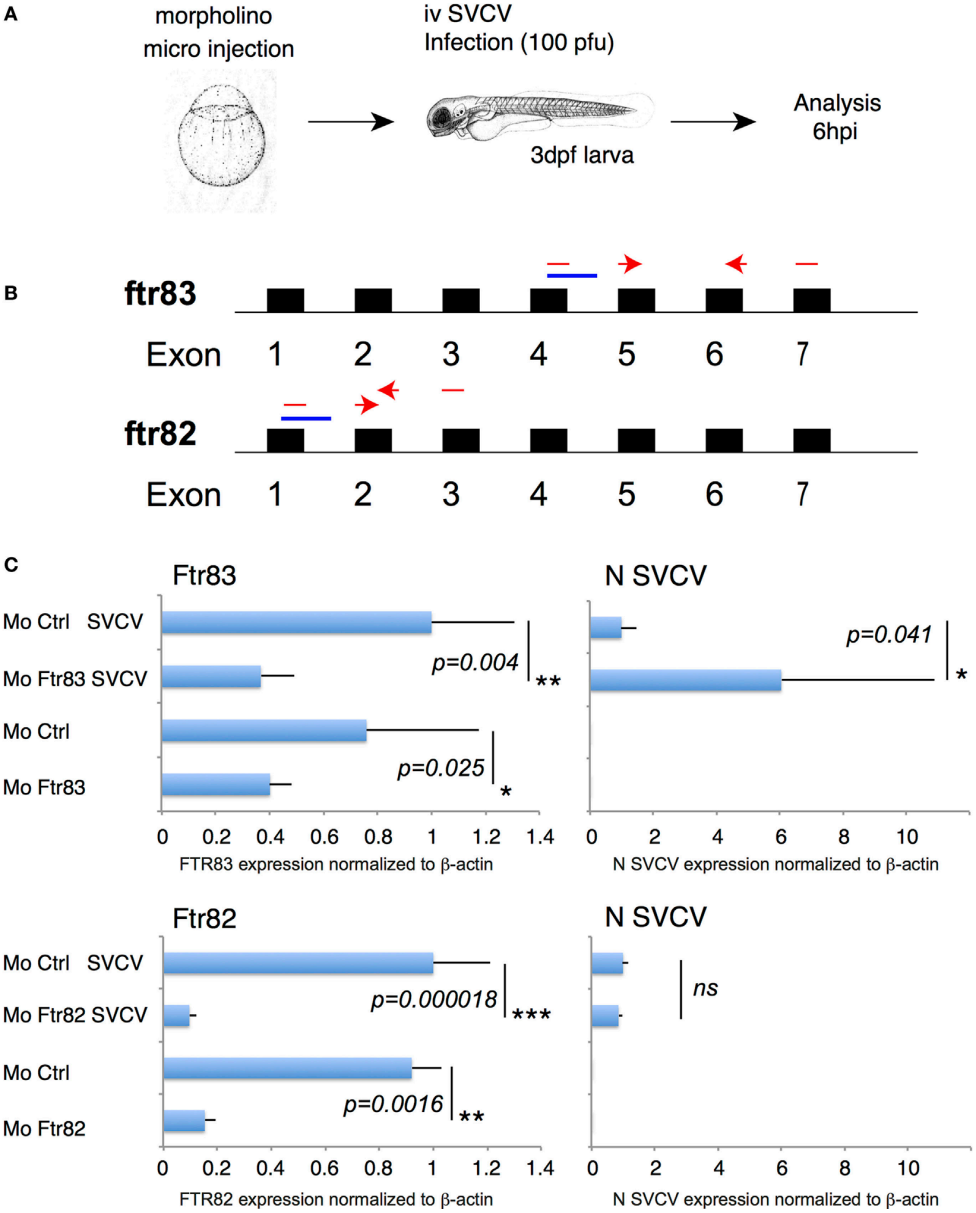
**Ftr83 exerts antiviral activity *in vivo* in zebrafish embryo**. **(A)** Schematic drawing of the loss of function experiment in zebrafish embryos. Embryos were injected at the one-cell stage with 4 ng of the ftr83, ftr82 or control morpholino, injected at 72 hpi with 100 pfu of spring viremia of carp virus (SVCV), and analyzed 6 hpi. **(B)** Schematic representation of the location of splice *ftr83* and *ftr82* morpholinos in the respective genes (in blue). Primers allowing to distinguish between spliced and unspliced mRNA are shown in red. **(C)** The expression of *ftr83, ftr82*, and viral *N* transcripts was quantified 6 hpi by RT-PCR. Results were normalized on the β-actin expression. The larvae were injected with the ftr83 morpholino (MO Ftr83), with the ftr82 morpholino (MO ftr82), or with the control morpholino (MO Ctlr), and infected with SVCV infection or mock-infected. Data are shown relative to the expression level of the analyzed mRNA in larvae injected with the ftr83 morpholino (upper panels) or with the ftr82 morpholino (lower panels) and infected by SVCV (set to 1). Mean and SD are shown, for four independent experiments. Stars indicate significant differences using Student’s *t*-test (*p < 0.05).

To examine whether the robust antiviral effect of FTR83 overexpression in EPC cells discloses its natural function *in vivo*, we performed loss of function experiments in zebrafish embryos (Figure 7A). *ftr83* was knocked down using a splice-blocking morpholino (moFTR83), which allows for a quantification of knockdown efficiency by qRT-PCR (Figure 7B). As shown in Figure 7C, moFTR83 significantly decreased the expression of *ftr83* 72hpf, compared with the control morpholino. Embryos were then infected i.v. with SVCV, and their RNA prepared 6 h later. Transcripts encoding the N protein of the virus were indeed significantly more expressed in embryos in which *ftr83* expression had been knocked down, compared to controls (Figure 7C). Importantly, no difference in viral replication was observed when FTR82 was knocked down. These results confirm that the antiviral activity observed in EPC cells overexpressing FTR83 is also observed *in vivo*.

In keeping with this, we also observed that expression level of *ftr83* in gills of the adult, which is significantly variable from fish to fish, shows a fair correlation with the expression of *ifn*φ*1* (Figure S3C in Supplementary Material). While *ftr83* was not induced by IFN, this correlation supported the notion that FTR83 might also act *in vivo* through the modulation of type I IFN expression in surfaces exposed to pathogens from the environment.

## Discussion

Over the last decade, the TRIM family emerged as a subset of key factors involved in antiviral defense. Antiviral TRIMs have been involved in multiple types of mechanisms, and recent large-scale functional studies revealed that TRIMs are frequently modulators rather than direct effectors of antiviral immunity (21, 47). Since TRIM proteins are also involved in many basic cellular functions, whether their implication in antiviral mechanisms is a primordial feature of the family has remained an open question.

While TRIM genes are present across metazoans, the implication in antiviral immunity of the few *trim* genes found in the genomes of basal branches of metazoans or in invertebrates (5, 23, 24, 48) remains unknown. Interestingly, these *trim* mostly belong to the class 1 (domain structure: RBCC-COS-Fn3-B302), which enhances the RIG I pathway in human (21). In vertebrates, *trim* with a “RBCC-B30.2” domain structure (class 4) greatly diversified independently in several groups such as fish, crossopterygians, and mammals (25, 26). In fish, several such expansions can be observed in available genome sequences, e.g., TRIM35, TRIM39 (btr), and finTRIM (ftr) in the zebrafish (26). The large TRIM subset named “FinTRIM”, in particular, was suspected to play a role in antiviral immunity: these genes were discovered as virus- and IFN-induced genes and constituted a large and diverse group, in which the B30.2 domain evolved under positive selection throughout the diversification (28). Such signatures of positive (i.e., diversifying) selection in the loop corresponding to the viral binding site of primate TRIM5α (36) strongly suggest that they might directly bind diverse ligands, possibly viral epitopes. In contrast, a small subset of *ftr* displayed different features (28). They were expressed constitutively at higher levels compared to other *ftr* and were not induced by viral infection or type I IFN. Additionally, they were conserved across teleosts in contrast to the other *finTRIMs* genes, which apparently constitute independent expansions in different fish groups, for example, in cyprinids and salmonids. Thus, zebrafish *ftr83* and *ftr82* are at a “basal” position in the tree of *ftr* and likely are the closest zebrafish representatives of the primordial finTRIMs (28). The implication of these genes in immunity was therefore of particular interest to understand the evolution of species-specific mechanisms of antiviral TRIMs across vertebrates.

Our data showed that FTR83 mediates a strong antiviral activity against different RNA viruses, including enveloped and non-enveloped viruses, supporting a primary function of basal finTRIM in antiviral immunity. Overexpression of FTR83 triggers the IFN signaling pathway, as transiently FTR83-transfected cells showed upregulation of type I *ifn* itself, as well as various ISGs: *ddx58, irf7, irf3, stat1b, rsad2*, and *isg15*, which mediate antiviral mechanisms in fish and mammals (49, 50). FTR83-based protection apparently relies on the induction of IFN, since the establishment of an antiviral state perfectly correlated with the type IFN response. The transfection of a truncated dominant negative mutant of IRF3 was sufficient to inhibit both the FTR83-mediated antiviral activity and IFN induction, and that FTR83 acts upstream IRF3 in the pathway. Importantly, in the context of a viral infection, FTR83 promoted the IFN response that reached the same level in cells overexpressing FTR83, and in controls where the infection had developed much more. Altogether, our data show that immunomodulatory properties, a fundamental function of the TRIM family in mammals, are also associated to a fish finTRIM and represent a primordial feature of vertebrate TRIMs. Critically, the knockdown of *ftr83* in zebrafish embryo showed that this gene is indeed implicated in antiviral mechanisms *in vivo*. These data are particularly interesting in the context of a series of recent reports of positive or negative impacts on antiviral response for a number of TRIM proteins from a percomorph fish, the orange spotted grouper (29–34, 51). The impact of multiple fish TRIM proteins on antiviral immunity evokes a complex array of multiple factors with effects at many levels of immune pathways: both conserved *trim* (e.g., *trim25* or *trim32*) and fish-specific expansions (e.g., *fintrim* or *btr*) will provide beautiful models to understand the evolution of TRIM-mediated mechanisms.

The diversity of TRIM mechanisms and their specialization against different types of viruses suggested they might have site- or tissue-specific expression and participate to the regionalization of immunity. *ftr83* has a restricted pattern of expression to gills, skin, pharynx, and to a much lesser extent hematopoietic tissues, as shown by ISH in the larva and by QPCR on dissected tissues in the adult. We also noted that the level of *ftr83* expression in gills was highly correlated to the IFNφ1 expression in adult fish (Figure S3C in Supplementary Material), suggesting that ftr83 might act locally in areas constantly exposed to pathogens. Importantly, *ftr83* was expressed at a higher level than other finTRIMs—that are generally almost undetectable in non-infected animals [(43) and unpublished data]—and was not induced by IFN, suggesting that its pattern of expression in healthy fish may reflect the regions in which it exerts its antiviral activity. The tissue specificity of *ftr83* contribution to antiviral immunity would warrant further investigations.

The closest relative of *ftr83* in the zebrafish genome is *ftr82*, but its function—which remains to be understood—is clearly very different: in contrast to *ftr83, ftr82* overexpression did not afford protection of transfected cells against RNA viruses we tested and failed to induce any detectable IFN upregulation either upon basal conditions (FTR82 overexpression) or after short exposure of transfected cells to the RNA vesiculovirus SVCV. Indeed, a recent publication suggests that ftr82 plays a developmental role in vascular patterning (39). As *ftr82* and *ftr83* are closely related paralogs, their contrasted functional properties constituted a good system to investigate which domain(s) was responsible for the antiviral activity of FTR83 by exchanging the domains and making chimeric proteins. This approach has been previously used to demonstrate the important role of TRIM5α B30.2 domain to mediate antiretroviral activity (52). None of the FTR chimeras in which B30.2 domains had been swapped between FTR82 and FTR83 did afford protection against VHSV or IHNV, or modulation of IFN signaling pathway. Altogether, our data indicate that FTR83 antiviral mechanism required both its particular RING and B30.2 domains, as previously reported for several mammalian TRIMs (21, 47, 53–55). While RING domain supports E3 ubiquitin ligase activity and determines the specificity of the E2 conjugase (56), the selection of target proteins generally occurs through the C-terminal domain (10). This scheme is also consistent with the E3 ubiquitin ligase activity of finTRIM we showed previously (57).

In this work, we demonstrate that a member of the largest TRIM expansion, the zebrafish finTRIM, constitutes a potent amplifier of the type I IFN expression, and yields an antiviral activity against several viruses. It will be interesting to characterize at which level of the type I signaling pathway *ftr83* is involved, and if it may also affect other pathways. This work is also a first step toward the immune function of *ftr* genes. The diversity of finTRIM, like the one of other fish genes involved in immunity such as ISG (43), complement factors (58), or heparan sulfate producing enzymes (59), provides many opportunities for sub-functionalization and will likely require systematic screening approaches.

These findings provide a framework to understand the repeated TRIM gene expansions during vertebrate evolution, and support the notion that modulation of immune pathway is a primordial function of TRIM proteins. Since ftr83 has orthologs in other fish species, including species important in aquaculture, our data might be useful to identify relevant markers for selection of fish more resistant to viral diseases.

## Ethics Statement

All animals were handled in strict accordance with good animal practice as defined by the European Union guidelines for the handling of laboratory animals (http://ec.europa.eu/environment/chemicals/lab_animals/home_en.htm) and by the Regional Paris South Ethics committee. All animal work was approved by the Direction of the Veterinary Services of Versailles (authorization number 78-28) as well as fish facilities (authorization number B78-720). Experimental protocols involving zebrafish were approved by the INRA institutional ethical committee “Comethea” (#12/114).

## Author Contributions

CL, J-PL, and PB designed the experiments; wrote the article. CL, EA, AH, CT, and AL performed the experiments. CL, EA, AH, J-PL, and PB analyzed the data.

## Conflict of Interest Statement

The authors declare that the research was conducted in the absence of any commercial or financial relationships that could be construed as a potential conflict of interest.
