# Supplementary material for: FTR83, a Member of the Large Fish-Specific finTRIM Family, Triggers IFN Pathway and Counters Viral Infection
Source: Front Immunol. 2017 May 26;8:617. doi: 10.3389/fimmu.2017.00617 (PMC5445110; doi:10.3389/fimmu.2017.00617)
Supplement: Supplementary file 1 [file Table_1.pdf]

Table S1 Primers used in this study.

	Forward primers	Reverse primers	Product size (nt)
WISH			
Ftr83 (CDS: 731-1550)	AATTAACCCTCACTAAAGGGAGAT CTCAAGCTGGTCTTTGTTG	TAATACGACTCACTATAGGGAGACCA TTGAAAGAATGCAGCAGG	821
Ftr82 (CDS: 740-1548)	AATTAACCCTCACTAAAGGGAGAAC TAACAGCCTTGTCTGCCC	TAATACGACTCACTATAGGGAGACTA TCGAGAGGATGAGGTCAG	809
RT QPCR*			
Ftr83	CGGAAATGCAGCAACGGCT	CTGAGTTCTTAAGAGCATCCA	113
Ftr82	GAGGGGAAAAACAGAAACTTC	TGCAGAGCCTTTGAGAGAGT	118
FTR83*	AGTGGCAAAGACAGTTAACAAA	TGGCAGGCATACTGAAGGAA	161
FTR82*	GAGGGGAAAAACAGAAACTTC	TGCAGAGCCTTTGAGAGAGT	119
SVCV-N	GATTGGGATTCAGGGAGAGA	AGCAAAGTCCGGTATGTAGT	185
rsad2	AGCGAGGCTTACGACTTCTG	GCACCAACTCTCCCAGAAAA	115
ddx58	TGCTGGACCGGATGTGTTATCT	TGGTGATCGATGGTTCGATTCT	177
ifnphi1	ATGAAAACTCAAATGTGGACGTA	GATAGTTTCCACCCATTTCCTTAA	147
myd88	CTGAAGCTTTGTGTGTTTGAC	ACCACCATCCTCTTACACCT	78
trif	TGGCGTACAGGAGAGCCCTT	GCCACCTGCGGCCCGCAGAC	136
tbk1	TGCTGGTGATGGAGTACTGC	ATGTTCCCTGGCTTGATGTCTC	174
rip1	TGGCTCGTAAACTGGGTCTG	CAGTTTACCCACGGTGATGCC	140
irf7	GAGCAAATACGCTTCCCGA	CTTGTCCTGACGAAAGCCATA	140
crfb5	CTAGGTCCTCCGTCCAGTGTGA	CATGTCCTGCACCAGCGTCTTC	113
jak1	GCCAGGATCTCTGGTATGCTCC	CCATGCCAGTTTGTGAAATAAA	102
irf9	AATCCCTGCTACCCTTCATG	AAGACCCGTCCCTGGCAGAA	90
stat1a	AGGCTGAGCTTTGCTGGTCT	TCAGCATGTTGTACCACAT	125
stat1b	GTGGAAGAAGAGACAGCAGAT	GTGAACCAGTTCTGCAGTTG	77
stat2	TTCAGGTAAGGATTCTCCTCTC	CGGATCATGGTAGCAGAAAA	103
irf3	AGTTATCCTGGAGTGTGTTGGACC	AAGTACCATCTTGAAGCCTTTG	219
Mxa	GGAGAATCAGTTACAAAACCT	GATTGTCTCTTGCCTTTGTAACA	
isg15	AACTCGGTGACGATGCAGC	TGGGCACGTTGAAGTACTGA	123
b-actin	GGGAGAAGATGACCCAGATCATG	GGTGGTACGGCCAGAGGC	97

* The specificity of qPCR amplification with primer pairs listed below has been checked by sequencing of products
** discrimination between spliced and unspliced isoforms

Cloning of FTR83		
FTR83-HA	ATGTCGTATGACCAGGACCTCTGTTACC	CTAAGCGTAATCTGGAACATCGTATGGG TACGATCCCGATCCCAGCTGACAAATAG TAACAGCAGACTCG
FTR83-Attb1	GGGGACAAGTTTGTACAAAAAAGCAGGC TTCGAAGGAGATAGAACCATGTCGTATGA [illegible]	cloning in gateway pDONR
FTR83-stop-Attb2	GGGGACCACTTTGTACAAGAAAGCTGGG TCTCAGAGCTGGTTGGACATTTGGTGGT G	cloning in gateway pDONR
FTR83-nostop-Attb2	GGGGACCACTTTGTACAAGAAAGCTGGG TCGAGCTGGTTGGACATTTGGTGGTG	cloning in gateway pDONR

FTR83 mutants		
Ftr83ΔB30.2-Attb2	GGGGACCACTTTGTACAAGAAAGCTGGGTCTACTGAAGGAACTCGTCTCTG	cloning in gateway pDONR
FTR83B30.2-Attb1	GGGGACAAGTTTGTACAAAAAAGCAGGCTTCGAAGGAGATAGAACCATGGCCTGCCAGCTTACTCTGGACC	cloning in gateway pDONR
FTR83B30.2-	GGGGACCACTTTGTACAAGAAAGCTG	cloning in gateway pDONR

**FTR82**		
Ftr82-Attb1	GGGGACAAGTTTGTACAAAAAAGCAGG CTTCGAAGGAGATAGAACCATGGCTGA GCAAATGTCTCCAC	cloning in gateway pDONR
Ftr82-stop-Attb2	GGGGACCACTTTGTACAAGAAAGCTGG GTCTCATTTCAATTGGCAGATCTTGA	cloning in gateway pDONR
Ftr82-nostop-Attb2	GGGGACCACTTTGTACAAGAAAGCTGG GTCTTTCAATTGGCAGATCTTGAC	cloning in gateway pDONR
HA-Ftr82	ACCATGTACCCATACGATGTTCCAGAT TACGCTGGATCGGGATCGGCTGAGCAA ATGTCTCCAG	TCATTTCAATTGGCAGATCTTGAC
**Chimeras**		
Start B30.2 ftr82	GAGTTCCTTCAGTATTACTGTCAGCTG AAA	
End RBCC-ftr82	AGTAAGCTGGCAGGCGTATTTCAGAAAC TC	
Start B30.2 ftr83	GAGTTTCTGAAATACGCCTGCCAGCTT ACT	
End RBCC-ftr83	TTTCAGCTGACAGTAATACTGAAGGAAC TC	
