# Supplementary figures and images for: FTR83, a Member of the Large Fish-Specific finTRIM Family, Triggers IFN Pathway and Counters Viral Infection
Source: Front Immunol. 2017 May 26;8:617. doi: 10.3389/fimmu.2017.00617 (PMC5445110; doi:10.3389/fimmu.2017.00617)

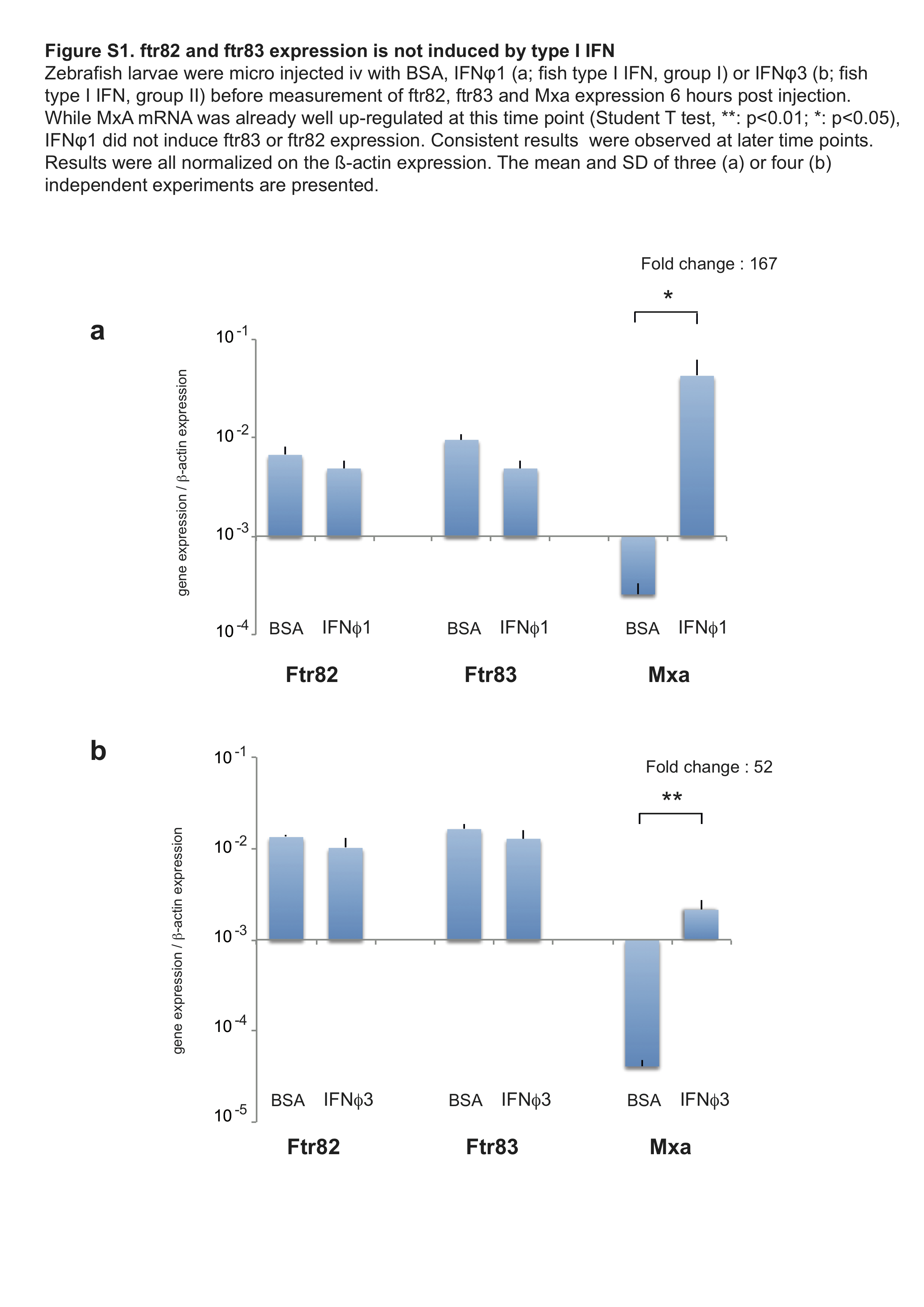

Supplement: Supplementary file 2 [file Image_1.tiff]

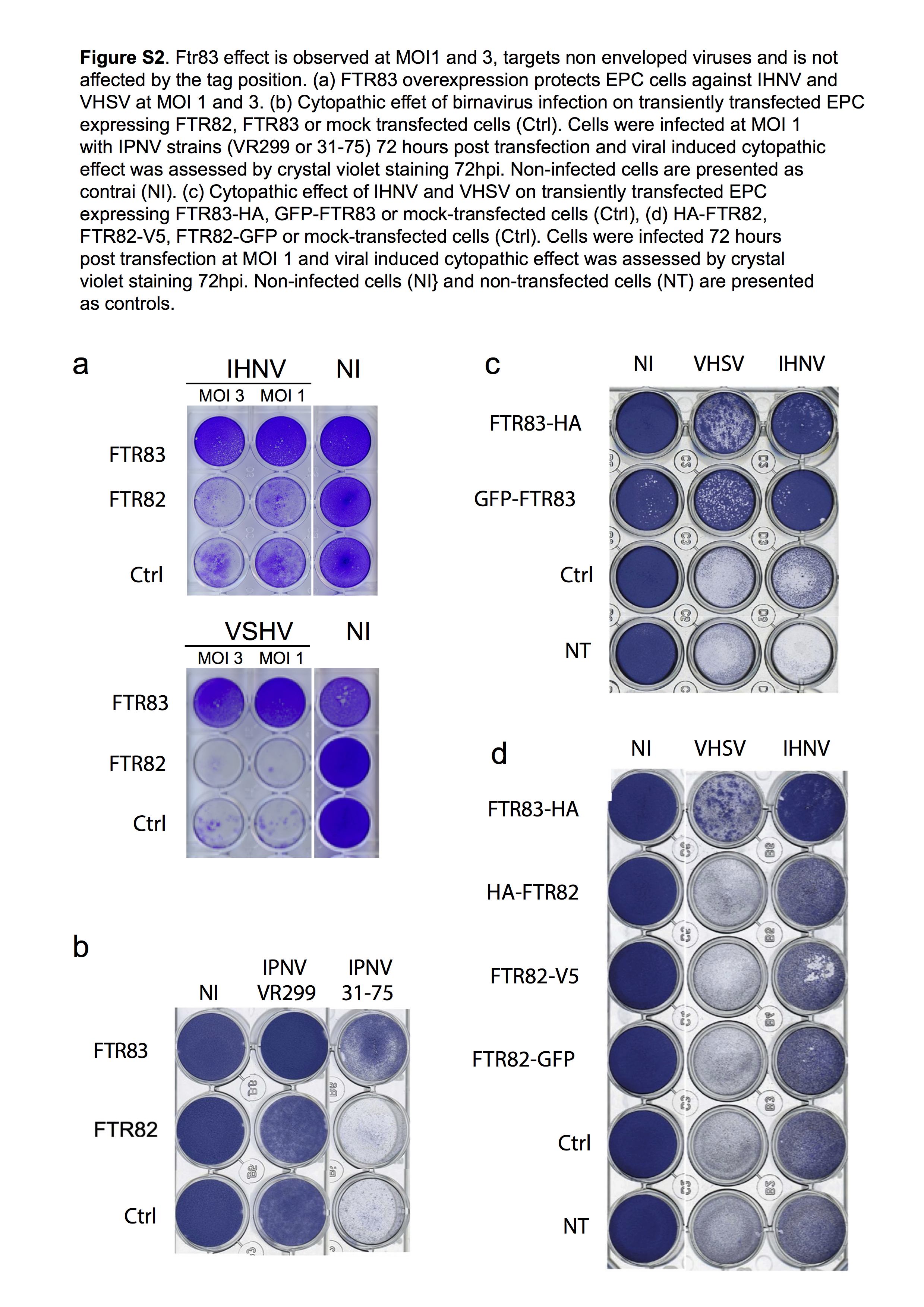

Supplement: Supplementary file 3 [file Image_2.tiff]

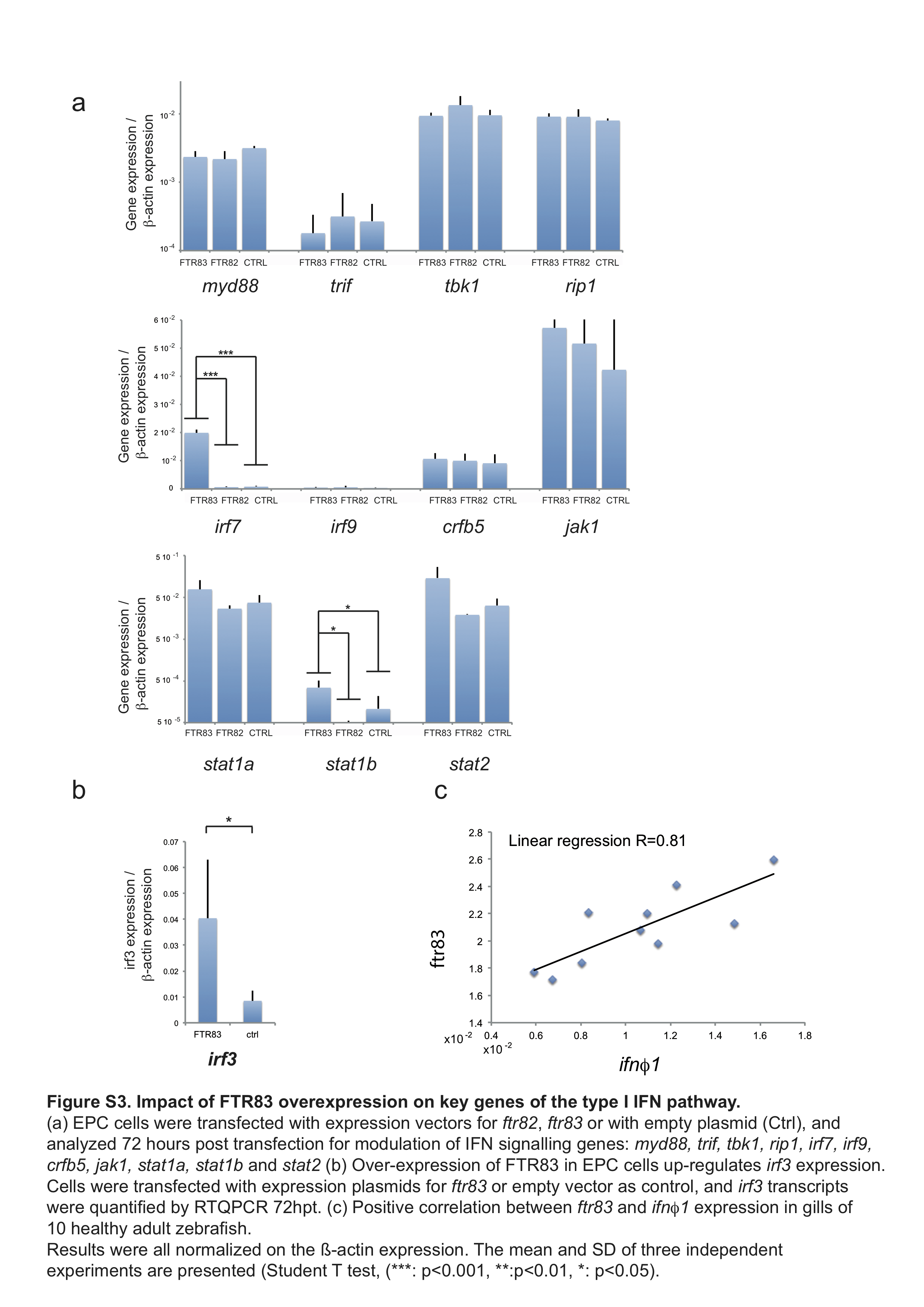

Supplement: Supplementary file 4 [file Image_3.tiff]

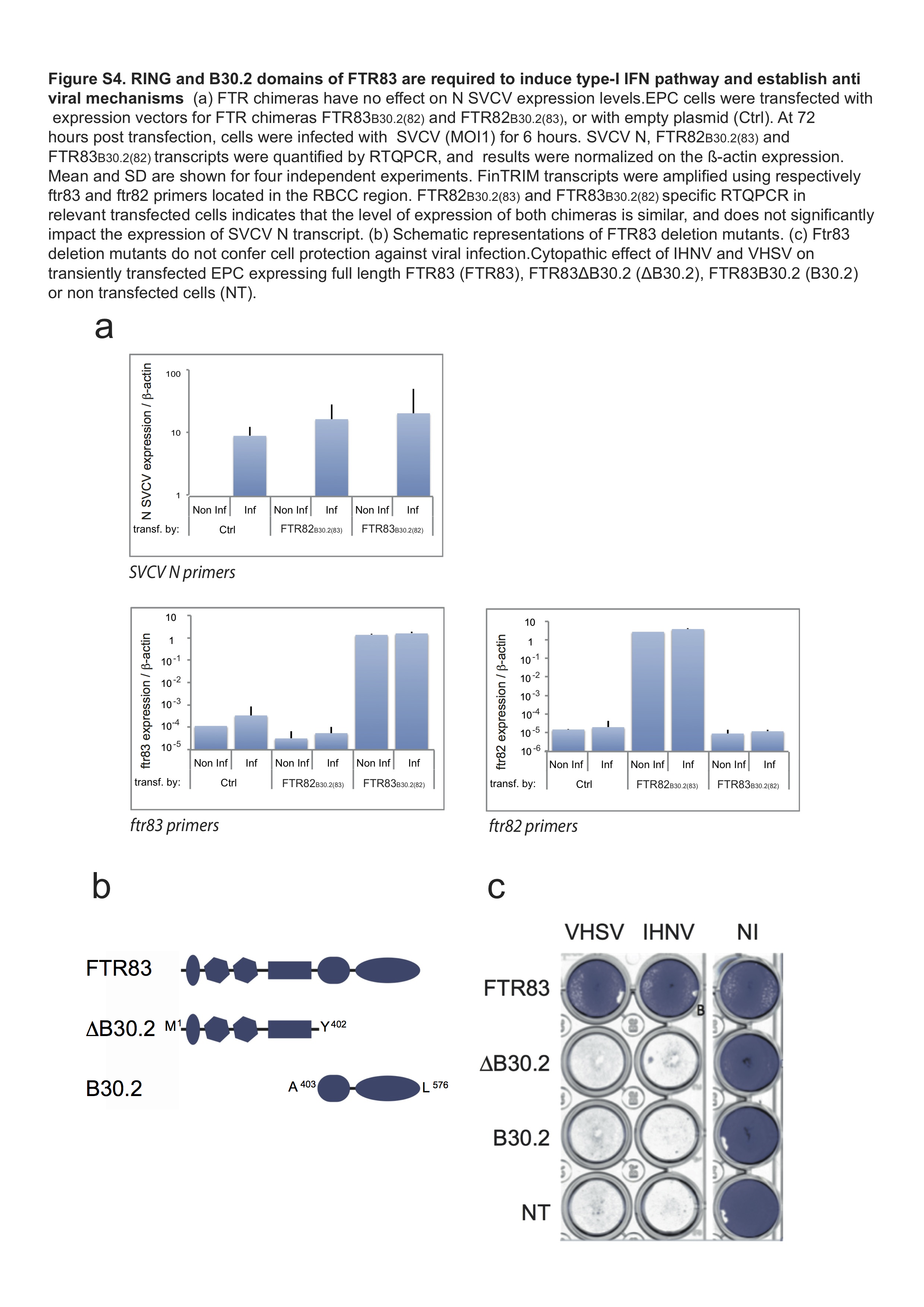

Supplement: Supplementary file 5 [file Image_4.tiff]
